# ERBB2 Regulates MED24 during Cancer Progression in Mice with *Pten* and *Smad4* Deletion in the Pulmonary Epithelium

**DOI:** 10.3390/cells8060615

**Published:** 2019-06-19

**Authors:** Jian Liu, Tianyuan Wang, Cynthia J. Willson, Kyathanahalli S. Janardhan, San-Pin Wu, Jian-Liang Li, Francesco J. DeMayo

**Affiliations:** 1 Reproductive & Developmental Biology Laboratory, National Institute of Environmental Health Sciences (NIEHS), Research Triangle Park (RTP), NC 27709, USA; steve.wu@nih.gov; 2 Integrative Bioinformatics Support Group, NIEHS, RTP, NC 27709, USA; tianyuan.wang@nih.gov (T.W.); jianliang.li@nih.gov (J.-L.L.); 3 Integrated Laboratory Systems, Morrisville, NC 27560, USA; cynthia.willson@nih.gov (C.J.W.); kyathanahalli.janardhan@nih.gov (K.S.J.)

**Keywords:** ERBB2, MED24, lung cancer, *Pten*, *Smad4*, EGFR

## Abstract

ERBB2 is an oncogenic driver with frequent gene mutations and amplification in human lung tumors and is an attractive target for lung cancer therapy. However, target therapies can be improved by understanding the in vivo mechanisms regulated by ERBB2 during lung tumor development. Here, we generated genetic mouse models to show that *Erbb2* loss inhibited lung tumor development induced by deletion of *Pten* and *Smad4*. Transcriptome analysis showed that *Erbb2* loss suppressed the significant changes of most of the induced genes by ablation of *Pten* and *Smad4*. Overlapping with ERBB2-associated human lung cancer genes further identified those ERBB2 downstream players potentially conserved in human and mouse lung tumors. Furthermore, MED24 was identified as a crucial oncogenic target of ERBB2 in lung tumor development. Taken together, ERBB2 is required for the dysregulation of cancer-related genes, such as *MED24*, during lung tumor development.

## 1. Introduction

Lung cancer is the leading cause of cancer death in the United States and worldwide [1,2]. Non-small cell lung cancer (NSCLC) accounts for approximately 85% of all cases and is the most common subtype of lung cancer [1]. NSCLS is further divided into adenocarcinoma, squamous cell carcinoma (SCC), and large cell carcinoma [1,2]. These tumor subtypes also have distinct morphologies and molecular profiles [3,4]. The diversity of lung tumor subtypes makes it impossible for one drug to be effective on all lung cancer cell types [2]. Therefore, understanding the molecular profiles of lung tumors will significantly benefit lung cancer targeted therapy.

One target for lung cancer therapy is members of the Epidermal Growth Factor Receptor (EGFR) family. This family consists of four members: EGFR, ERBB2, ERBB3, and ERBB4 [5,6]. All four members have a similar structure, with an extracellular ligand binding domain, a transmembrane domain, and an intracellular domain interacting with a multitude of signaling molecules [7,8]. They have both ligand-dependent and ligand-independent activity [7,8]. Of note, there is no ligand identified for ERBB2 [7,8]. In fact, ERBB2 works as the preferred dimerization partner of the other EGFR receptors [9] and can be activated in diverse manners, such as by phosphorylation, gene expression, and mutations [10,11,12]. These receptors, as oncogenes, were used as therapeutic targets for numerous cancers, including lung cancer, colorectal cancer, breast cancer, and glioblastoma [13,14]. ERBB2 was validated as an efficient target in breast cancer therapy using its monoclonal antibody Trastuzumab (marketed as Herceptin) [15]. Meanwhile, additional studies have shown that ERBB2 is a critical driver gene in human lung cancer and is frequently altered in the development of lung cancer by gene mutations and amplification [16,17]. Lung cancer patients with ERBB2 activation have been treated using its kinase inhibitor (e.g., Lapatinib) or its mono-antibody (e.g., Trastuzumab). However, these treatments have limited efficacy, and patients often develop resistance to these drugs [18]. Therefore, more studies are needed to understand the underlying mechanism of ERBB2-regulated lung cancer development.

In vivo models have served to shed light on the investigation of the role of genes (e.g., *ERBB2*) in the development and progression of lung cancer. Overexpression of wild-type human *ERBB2* in mouse lungs was shown to cause the development of adenocarcinoma [19]. Overexpression of the most common human ERBB2 mutant (*ERBB2*^YVMA^) in mice was shown to drive the development of lung adenosquamous tumors [20]. However, overexpression models do not show the physiological role of ERBB2 in the development of lung cancer. We have previously shown that ERBB2 phosphorylation was induced in mouse lung adenosquamous carcinoma caused by the pulmonary epithelial deletion of *Pten* and *Smad4* [12]. In this study, we investigated the physiological significance of ERBB2 activation in this model by ablating *Erbb2* in the lungs of mice with *Pten* and *Smad4* deletion. The impact of *Erbb2* ablation on lung tumor development in the *Pten*^−/−^/*Smad4*^−/−^ model demonstrated that the *Erbb2* gene was required for lung tumor development. Microarray analysis was used to determine the transcriptome profiles regulated by *Erbb2* during mouse lung tumor development. Our analysis demonstrated that ERBB2 regulated the expression of one of the transcriptional mediator complex (MED) genes (e.g., *MED24*). Taken together, our in vivo genetic evidence shows ERBB2 to be essential for lung tumor development and integrated analyses established a potential link between the EGFR family and the MED genes in lung tumor development.

## 2. Materials and Methods

### 2.1. Mice

*CCSP*^iCre^, *Pten*^f/f^, *Smad4*^f/f^, and *Erbb2*^f/f^ mice are described in previous publications [12,21]. All animal protocols were approved by the An Institutional Animal Care and Use Committee (IACUC) at the National Institute of Environmental Health Sciences and Baylor College of Medicine. All experiments were conducted in accordance with relevant guidelines and regulations of both institutions. All mice were B6; 129 backgrounds and genotyped by Transnetyx. Female and male mice were purposely included in all the mouse experiments. No differences were observed between female mice and male mice in lung tumor development.

### 2.2. Cell Derivation, Culture, and siRNA Knockdown

H358 cell line was purchased from the American Type Culture Collection (ATCC) and cultured using Roswell Park Memorial Institute (RPMI) and 10% fetal bovine serum (FBS), Penicillin (100 IU/mL), and Streptomycin (100 μg/mL) according to protocol. Before the cells were treated with EGF, the cells were starved using only RPMI overnight. A 50 nM siRNA smart pool from Dharmacon was used as the final concentration to knockdown *MED24* or non-targeting genome sites using Lipofectamine RNAiMAX Reagent (Cat.: 13778150, ThermoFisher Scientific, Waltham, MA, USA) following the manufacturer’s protocol.

The siRNAs sequence for *MED24* (L-021247-00-0005) is CAACCUGGCAGAUGCGUUA, CAAACAUCCUCAAGACGAU, GUACGGAGGAGCUCAAGUG, and CCAAUCCUCUCAUCUUGUC; and the siRNAs sequence for the control (D-001810-10-05) is UGGUUUACAUGUCGACUAA, UGGUUUACAUGUUGUGUGA, GGUUUACAUGUUUUCUGA, and UGGUUUACAUGUUUUCCUA.

### 2.3. Cell Proliferation

Approximately 2000 H358 cells were seeded in replicates of five for each group on a 96-well plate at Day 0. Twenty microliters (20 uL) of MTS reagent (Promega, Madison, WI, #G3582) was added for each assay into each well containing 200 uL of cell culture medium, incubated for 1 h, and then analyzed at an absorbance at 490 nm with a 96-well plate reader.

### 2.4. Histopathology and Immunohistochemistry

Mouse lungs and lung tumors were fixed in 4% paraformaldehyde and paraffin-embedded following previous immunohistochemistry (IHC) methods [12,22]. Antigen Unmasking Solution (Citric Acid Based from Vector Laboratories, H-3300) was used for the antibody incubation: MED24, TTF1, P63, and CK5. Antigen Unmasking Solution was used to incubate slides with EDTA-TE buffer (1 mM, PH 8.0, 0.1% Tween-20) for 25 min under 100 °C; and then TE buffer (10 mM Tris-HCL, PH 9.0, 1 mM EDTA, 0.1% Tween-20 under 100 °C for 25 min) was used for ERBB2 antibody incubation. Normal goat serum and an Avidin/Biotin Blocking Kit (Vector, SP-2001) were used for blocking the slides before the primary antibody incubation. These antibodies included ERBB2 antibody (Santa Cruz Biotechnologies, Dallas, TX, sc-284, 1:2000 dilution), MED24 (Sigma-Aldrich, St. Louis, MO, USA, SAB4503717-100UG, 1:2000 dilution), TTF1 antibody (DAKO, Santa Clara, CA, USA, M3575, 1:2000 dilution), P63 (Cell Signaling, Danvers, MA, USA, 13109, 1:1000 dilution), and CK5 antibody (Abcam, Cambridge, MA, USA, ab52635, 1:3000 dilution). A 1:400 dilution was used for the secondary antibodies, such as biotinylated goat anti-rabbit IgG antibody (Vector Laboratories, Inc., Burlingame, CA, USA, BA-1000) and biotinylated goat anti-mouse igg antibody (Vector Laboratories, Inc., Burlingame, CA, BA-9200). An ABC kit (Vector Laboratories, Inc., Burlingame, CA, PK-6100) was used to amplify the signaling and then a DAB kit (Vector Laboratories, Inc., Burlingame, CA, SK-4105) was used for detection of final positive signaling (brown staining).

### 2.5. RNA Isolation, qRT-PCR, and Microarray Analysis

#### 2.5.1. RNA Isolation

Using the protocol for RNA isolation previously published [12,22], total RNAs were isolated from mouse lungs or lung tumors using TRIzol reagent and cleaned utilizing the RNeasy kit (Qiagen, Germantown, MD, USA, Cat.: 74104). Total RNAs were isolated from human cells using the RNeasy kit.

#### 2.5.2. qRT-PCR

The SYBR green system was used here following the protocol previously published [12,22]. The chosen housekeeping gene was 18s. The primer sequences were the following: *Erbb2* mouse primers (Forward primer 5′-3′: GAGACAGAGCTAAGGAAGCTGA; Reverse primer 5′-3′: ACGGGGATTTTCACGTTCTCC); *Med24* mouse primers (Forward primer 5′-3′: CACCCGAGCCAATCAACCAA; Reverse primer 5′-3′: ATGGTGCCCTCAAGCAAGATG); *MED24* human primers (Forward primer 5′-3′: GTCTGAGCTGTCACGGCAAA; Reverse primer 5′-3′: TGGTGCTGCTGAGGGTTTTC); and *18s* primers (Forward primer 5′-3′: GTAACCCGTTGAACCCCATT; Reverse primer 5′-3′: CCATCCAATCGGTAGTAGCG).

#### 2.5.3. Experiment Processes of Microarray Analysis

Microarrays were done at the National Institute of Environmental Health Sciences (NIEHS) using Affymetrix Mouse Genome 430 2.0 GeneChip^®^ arrays (Affymetrix, Santa Clara, CA, USA). One hundred nanograms (100 ng) of total RNA was amplified as directed in the Affymetrix 3’ in vitro transcription (IVT) Express kit protocol, performing the IVT reaction for 16 h. A total of 12.5 μg of amplified biotin-aRNAs were fragmented and 10 μg were hybridized to each array for 16 h at 45 °C in a rotating hybridization oven using the Affymetrix Eukaryotic Target Hybridization Controls and protocol. Array slides were stained with streptavidin/phycoerythrin utilizing a double-antibody staining procedure and then washed for antibody amplification according to the GeneChip Hybridization, Wash, and Stain Kit and user manual. Arrays were scanned in an Affymetrix Scanner 3000 and data were obtained using the GeneChip^®^ Expression Console Software using the MAS5 algorithm to generate .CHP files. The resulting data were processed using the OmicSoft Array Studio (version 9.0) software.

#### 2.5.4. Expression Array Analysis of Microarray Analysis

The CEL files were used to identify differentially expressed genes by the Genomics Suite Gene Expression workflow of the Partek software package (version 7.17) (Partek, Inc., St. Louis, MO, USA). The Robust Multichip Analysis (RMA) algorithm with quantile for normalization and the log2 transformation was applied to generate signal values of all samples. Differentially expressed genes were defined using the filters of ANOVA unadjusted *p*-value less than 0.01 and absolute fold change greater than 2. All experiments were performed in triplicate with independent pools of RNA.

#### 2.5.5. Functional Analysis of Differentially Changed Genes (DEGs)

Functional analysis of DEGs was performed using the Ingenuity Pathway Analysis (IPA, www.ingenuity.com) based on the content (version: 44691306; Release Date: 2018-06-14).

#### 2.5.6. Heatmap

A heatmap of differentially expressed genes was generated by the Genomics Suite of the Partek software package (version 6. 7.17) (Partek, Inc., St. Louis, MO, USA). The normalized and log2 transformed expression values were standardized by shifting the mean to 0 and scaling the standard deviation to 1.

### 2.6. Western Blot

Western blot experiments were conducted following a previously published protocol [23]. These antibodies included the following: ERBB2 antibody (Santa Cruz Biotechnologies, Dallas, TX, USA, sc-284, 1:500 dilution); p-ERBB2 antibody (Santa Cruz Biotechnologies, Dallas, TX, sc-293110, 1:500 dilution); MED24 antibody (Sigma-Aldrich, St. Louis, MO, USA, SAB4503717-100UG, 1:4000 dilution); β-actin antibody (Sigma-Aldrich, St. Louis, MO, A5316, 1:4000 dilution). A 1:4000 dilution was used for the secondary antibody: Goat Anti-Rabbit IgG (Sigma-Aldrich, St. Louis, MO, A0545-1ML) and Goat Anti-mouse IgG (Sigma-Aldrich, St. Louis, MO, A9044-2ML).

### 2.7. Correlation Analysis and Survival Analysis

The cBioPortal (http://www.cbioportal.org/) database was used. Here, we specifically used two datasets (Lung adenocarcinoma, The Cancer Genome Atlas (TCGA), Provisional; Lung squamous cell carcinoma, TCGA, Provisional). The default setting of the correlation analysis was used to obtain these *ERBB2*-correlated genes. The spearman correlation method was taken, not including mutation profiles. For the separation of lung cancer patients based on MED24 mRNA expression, an mRNA Expression z-Scores (RNA Seq V2 RSEM) threshold of ±2.5 was used. Consequently, 377 patients were qualified for the Kaplan–Meier survival analysis; 20 of them were considered to be in the MED24 high expression group while the rest were in the MED24 low expression group.

### 2.8. Quantification and Statistical Analysis

GraphPad Prism (version 7.02) was used for statistical analysis. Data are expressed as mean +/− SD. The sample size (*n*) represents biological replicates. Student’s *t*-test was used for comparison of two group averages. Statistical significance was considered for all of the datasets when the *p*-value was less than 0.05. All the bioinformatics and statistical analyses were analyzed or confirmed by bioinformaticians at NIEHS. All histopathological results were evaluated and provided by pathologists at NIEHS. The group size was determined based on the results of preliminary experiments and no statistical method was used to predetermine the sample size in animal studies. The genotyping of mice was conducted by Transnetyx Company (Cordova, TN, USA). Microarray experiments were unbiasedly conducted and analyzed by core facilities at NIEHS.

### 2.9. Data Availability

Array data were deposited in the Gene Expression Omnibus database (GEO, accession GSE128620).

## 3. Results

### 3.1. In Vivo Ablation of Erbb2 Prevents Pten^−/−^Smad4^−/−^-Induced Lung Tumor Development

**Figure 1:**
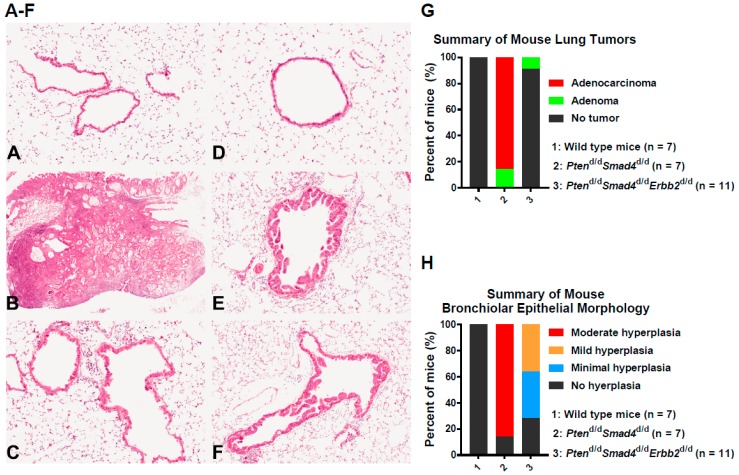
In vivo ablation of *Erbb2* prevents lung tumor development induced by compound deletion of *Pten* and *Smad4*. (**A**–**F**). Hematoxylin and eosin (H&E) staining of mouse lungs and lung tumors at 12 to 13 months of age. (**G**–**H**). Quantification of tumor incidences (**G**) or hyperplasia (**H**) in mouse lungs from the mice shown in Figures A–F and listed in Table S1. Pulmonary bronchioles from two wild-type mice (Figure 1A,D) are lined by a single layer of simple columnar epithelium. A representative pulmonary adenocarcinoma, which was present in almost all of the *Pten*^d/d^*Smad4*^d/d^ mice, is shown in Figure 1B. In addition to adenocarcinomas, most *Pten*^d/d^*Smad4*^d/d^ mice also exhibited moderate hyperplasia of the bronchiolar epithelium (Figure 1E), seen diffusely throughout the lung as the proliferation of the epithelial cells circumferentially lining the bronchioles, with crowding and outfolding or short papillary projections into the bronchiolar lumen. In contrast, *Pten*^d/d^*Smad4*^d/d^*Erbb2*^d/d^ mice did not have adenocarcinomas, and either did not have bronchiolar epithelial hyperplasia or, if present, it was either minimal (Figure 1C) or mild (Figure 1F) in severity. Minimal bronchiolar epithelial hyperplasia was seen as a slight crowding of epithelial cells in occasional bronchioles. Mild bronchiolar epithelial hyperplasia was seen as some crowding and outfolding, but with uneven distribution both within single bronchioles and throughout the lung.

Our previous study determined that *Pten*^d/d^*Smad4*^d/d^ (*CCSP*^iCre^*Pten*^d/d^*Smad4*^d/d^) mice developed lung tumors at 1 year of age with 100% incidence by activation of the ERBB2 pathway [12]. Here, we generated *Pten*^d/d^*Smad4*^d/d^*Erbb2*^d/d^ (*CCSP*^iCre^*Pten*^d/d^*Smad4*^d/d^*Erbb2*^d/d^) mice to examine whether *Erbb2* ablation inhibited lung tumor development induced by deletion of *Pten* and *Smad4*. In comparison with the wild-type group, all *Pten*^d/d^*Smad4*^d/d^ mice developed lung tumors (adenocarcinomas or, less frequently, adenomas) at 12 to 13 months of age while less than 10% of *Pten*^d/d^*Smad4*^d/d^*Erbb2*^d/d^ mice had tumor (adenomas) in the lungs at a similar age (Figure 1A–C,G). We previously reported that *CCSP*^iCre^ mice had strong Cre activities in lung bronchial epithelial cells [12]. Therefore, histopathological evaluation of bronchiolar epithelium was also conducted. In addition to lung adenocarcinomas, *Pten*^d/d^*Smad4*^d/d^ mice had bronchiolar epithelial hyperplasia of moderate severity, which was characterized by crowding and folding of the epithelium, with multiple layers and papillary projections into the bronchiolar lumen (Figure 1B, 1E, and 1H). In contrast, the bronchiolar hyperplasia seen in *Pten*^d/d^*Smad4*^d/d^*Erbb2*^d/d^ mice was either absent or less severe (of minimal to mild severity) when compared with *Pten*^d/d^*Smad4*^d/d^ mice (compare Figure 1C,E,F,H). The summary of pathological phenotypes (e.g., lung tumors and hyperplasia) for each mouse is described in Table S1. Taken together, genetic ablation of *Erbb2* in mouse lungs prevents malignant lung tumor development caused by deletion of *Pten* and *Smad4*.

### 3.2. Transcriptome Analysis Identifies ERBB2-Regulated Cancer Genes

**Figure 2:**
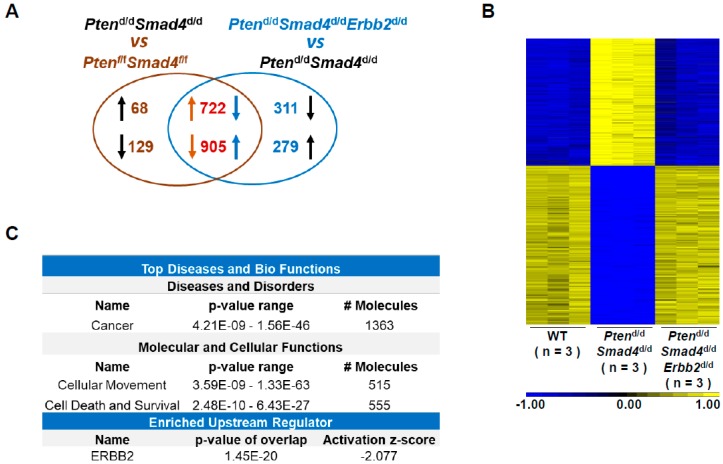
Transcriptome analysis of ERBB2-regulated genes associated with cancer development. (**A**) Overlapping analysis of differentially changed genes identified in microarrays using mouse lungs and lung tumors at 12 to 13 months of age. (**B**) Heatmap analysis of overlapped genes identified in Figure 2A. (**C**) Ingenuity Pathway Analysis of overlapped genes identified in Figure 2A.

Investigation of the transcriptome profiles modified by *Erbb2* loss during lung cancer development can help us to understand the molecular impact of ERBB2 on lung tumor formation. Here, we conducted microarray analysis of wild-type lungs, *Pten*^d/d^*Smad4*^d/d^ lung tumors, and *Pten*^d/d^*Smad4*^d/d^*Erbb2*^d/d^ lungs at 12 to 13 months of age. Two comparisons were conducted to extract the DEGs, whose cutoff was an unadjusted *p*-value less than 0.01 and an absolute fold change greater than 2. The first comparison was performed between *Pten*^d/d^*Smad4*^d/d^ lung tumors and wild-type lungs, identifying 790 upregulated genes and 1034 downregulated genes (Figure 2A and Table S2).

The second comparison was conducted between *Pten*^d/d^*Smad4*^d/d^*Erbb2*^d/d^ lungs and *Pten*^d/d^*Smad4*^d/d^ lung tumors, and we identified 1184 upregulated genes and 1033 downregulated genes (Figure 2A and Table S3). The analysis of overlapping DEGs found that most of the DEGs identified in the first comparison showed the reversed expression pattern in gene-altered *Pten*^d/d^*Smad4*^d/d^*Erbb2*^d/d^ lungs and *Pten*^d/d^*Smad4*^d/d^ lung tumors (Figure 2A and Table S4). The expression heatmap of these overlapped genes further demonstrates that *Erbb2* loss normalized the expression patterns of cancer-related genes (Figure 2B). Ingenuity Pathway Analysis (IPA) of these genes impacted by *Erbb2* ablation in the cancer model (Table S4) showed that they are significantly related to cancer, cellular movement, cell death, and survival (Figure 2C). These DEGs were analyzed to determine upstream regulators, which is a sub-method of IPA to identify the critical regulators enriched by its significantly changed downstream genes. As expected, ERBB2 is one of the top enriched upstream regulators (Figure 2C). Its activity was inhibited when the fold changes of DEGs were used in the second comparison (*Pten*^d/d^*Smad4*^d/d^*Erbb2*^d/d^ vs. *Pten*^d/d^*Smad4*^d/d^) (Figure 2C and Table S5). These results, at a molecular level, show that *Erbb2* loss suppressed the transcriptome profile changes induced by deletion of *Pten* and *Smad4*. This explains at the molecular level how *Erbb2* loss prevents *Pten^−/−^Smad4^−/−^*-induced lung tumor development on the side of a genome-wide gene expression analysis.

### 3.3. Conserved ERBB2-Downstream Genes (e.g., MED24) between Mouse and Human Lung Cancer

To identify these common downstream target genes of ERBB2 in lung cancer between mouse and human, ERBB2-correlated genes in human lung adenocarcinoma and squamous cell carcinoma (SCC) were determined from the TCGA Provisional Database [24]. These two datasets were used because immunohistochemical analysis showed that these tumors are not only positive for TTF1 (a typical adenocarcinoma marker), but also for P63 and CK5 (typical SCC markers) (Figure S1). Therefore, our gene expression correlation analysis included both lung adenocarcinoma and SCC. In TCGA database, 3504 genes showed a positive correlation with *ERBB2* in human lung adenocarcinoma with a *p*-value less than 0.05 (Table S6). Meanwhile, 3808 genes had a positive correlation with *ERBB2* in human lung SCC with the same cutoff of the *p*-value (Table S7). A total of 3415 and 3146 negatively correlated genes with *ERBB2* in lung adenocarcinoma (Table S8) and SCC (Table S9) were found, respectively. Overlapping analysis between *ERBB2*-correlated human genes and the second comparison (*Pten*^d/d^*Smad4*^d/d^*Erbb2*^d/d^ vs. *Pten*^d/d^*Smad4*^d/d^) was consequently conducted. The downregulated genes in the second comparison were overlapped with *ERBB2* positively correlated human genes, and vice versa. There were 154 genes showing an *ERBB2* positive correlation between mouse lung tumors and human lung adenocarcinoma (Table S10). Similarly, 149 genes were identified to be *ERBB2* positively correlated genes between human lung SCC and mouse lung tumors (Table S11). By overlapping these upregulated genes identified in the second comparison (*Pten*^d/d^*Smad4*^d/d^*Erbb2*^d/d^ vs. *Pten*^d/d^*Smad4*^d/d^) with these *ERBB2* negatively correlated genes, 117 and 87 genes were found in human lung adenocarcinoma (Table S12) and SCC (Table S13), respectively. These overlapped genes were *ERBB2*-downstream targets potentially conserved between mouse and human lung tumors.

**Figure 3:**
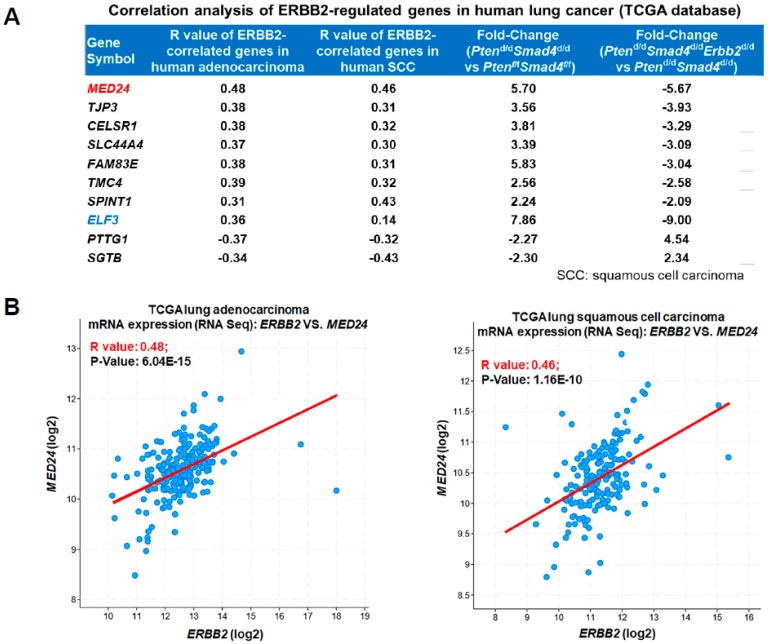
*MED24* is a conserved downstream target of *ERBB2* between mouse lung tumors and human lung cancer. (**A**) Top *ERBB2*-correlated genes in human lung adenocarcinoma and squamous cell carcinoma. (**B**) Correlation analysis between *MED24* and *ERBB2* in human lung adenocarcinoma (left panel) and squamous cell carcinoma (right panel).

We next identified candidate genes with significant changes in our mouse microarrays and with the highest correlation in both human lung adenocarcinoma and SCC (Figure S2 and Figure 3A). Among these top changed genes was *Med24* (Figure 3A). *MED24* not only had the highest correlation relationship (R-value) with *ERBB2* in lung adenocarcinoma and in SCC, but also had a significant fold change after *Erbb2* loss in mouse lungs (Figure 3A). The details of the correlation analysis between *MED24* and *ERBB2* in human lung cancer are included as an example for these correlated genes (Figure 3B). We hypothesized that these top *ERBB2*-correlated genes (Figure 3A), such as *MED24*, were crucial players regulating lung cancer development.

Other genes likely to be important for *ERBB2*-dependent lung tumor development uncovered by this correlation analysis were *ELF3* and *FAM83E.* We previously showed *ELF3* to be a key downstream oncogene of *ERBB2* in lung cancer development [12]. Here, it was identified in our correlation analyses (Figure 3A), suggesting it to be another contributor to *ERBB2*-dependent lung tumor development. *FAM83E* had a positive correlation with *ERBB2* in mouse and human lung tumors and also was overexpressed in mouse lung tumors (Figure 3A). In fact, the FAM83 family, including FAM83E, was reported to promote ERBB2 signaling [25]. Moreover, each FAM83 member was found to be overexpressed in at least one of 17 distinct tumor types, including lung cancer [26]. Of note, FAM83E promoted human mammary epithelial cell (HMEC) transformation [26] and was recently identified as an oncogene [25].

### 3.4. MED24 May Be an Oncogenic Player in ERBB2-Dependent Lung Cancer Development

**Figure 4:**
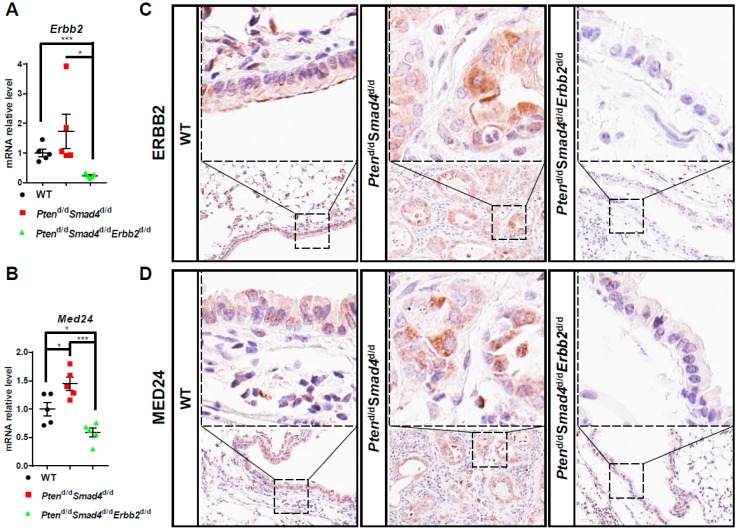
In vivo ablation of *Erbb2* in mice causes a decrease of *Med24* expression. (**A**,**B**) qRT-PCR analysis of mRNA expression of *Erbb2* (A) and *Med24* (B) in mice at 12 to 13 months of age (*n* = 5). Error bar using SD and *t*-test; * *p*-value < 0.05; *** *p*-value < 0.01. (**C**,**D**) Immunohistochemistry analysis of protein expression of ERBB2 (**C**) and MED24 (**D**) in mice at 12 to 13 months of age (*n* > or = 5). Scale bar (Bottom panel: black box (50 um)).

To validate the regulation of ERBB2 on MED24 expression in mouse lung tumors, we examined the expression of MED24 and ERBB2 in wild-type lungs, *Pten*^d/d^*Smad4*^d/d^ lung tumors, and *Pten*^d/d^*Smad4*^d/d^*Erbb2*^d/d^ lungs at 12 to 13 months of age. Consistent with the observation in the first and second comparisons in the microarray analyses (Tables S2 and S3), the mRNA expression of *Erbb2* and *Med24* was significantly downregulated after *Erbb2* loss (Figure 4A,B). Meanwhile, there was an increase of *Erbb2* and *Med24* mRNA expression after deletion of *Pten* and *Smad4* in comparison to the wild-type group (Figure 4A,B). In line with the mRNA expression results, the protein levels of ERBB2 and MED24 confirmed the mRNA and immunohistochemical analysis. (Figure 4C,D and Figure S3). These results suggest that *Med24* is a downstream gene of ERBB2 in mouse lung tumor development.

**Figure 5:**
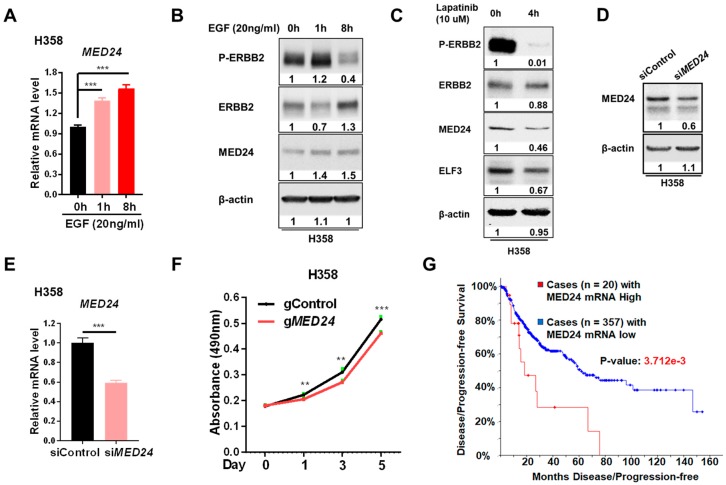
Knockdown of *MED24*, an epidermal growth factor (EGF) response gene, in human lung cancer cells attenuates cell growth. (**A**) qRT-PCR analysis of mRNA expression of *MED24* in H358 cells, a human lung adenocarcinoma cell line. Error bar using SD and *t*-test; ***, *p*-value < 0.01. (**B**,**C**) Western blot analysis of protein expression with the treatment of EGF (**B**) or Lapatinib (**C**). (**D**,**E**) Western blot (D) and qRT-PCR (E) analyses of protein (**D**) and mRNA (**E**) expression after knockdown of *MED24* using siRNA. Error bar using SD and *t*-test; ***, *p*-value < 0.01. (**F**) MTS assay of viable cells after knockdown of *MED24* using siRNA. MTS is a novel tetrazolium compound [3-(4,5-dimethylthiazol-2-yl)-5-(3-carboxymethoxyphenyl)-2-(4-sulfophenyl)-2H-tetrazolium, which is for cell proliferation assay. Error bar using SD and *t*-test; **, *p*-value < 0.01; ***, *p*-value < 0.01. (**G**) Survival analysis of lung cancer patients with relatively high or low expression of *MED24* using an online database. Image J was used to quantify the relative level of protein bands in Figure 5B–D; All experimental groups were compared to their treatment control groups.

Given that ERBB2 is an EFGR family member, it can be activated by its ligand treatment (e.g., EGF) [10,27]. We next asked whether EGF treatment could induce MED24 expression in human lung tumor cells. Upon EGF treatment, MED24 expression was induced at mRNA and protein levels (Figure 5A,B). In agreement with this induction result, inhibition of ERBB2 phosphorylation by its kinase inhibitor (e.g., Lapatinib) caused the decrease of MED24 expression (Figure 5C). These results (Figure 5A–C) suggest that MED24 is a downstream gene of the EGFR family in human lung cancer cells.

To examine whether MED24 expression is critical for the cell growth in human lung cancer cells, gene knockdown experiments were performed. We found inhibition of MED24 expression in human lung cancer cells (e.g., H358) using siRNA-reduced cell growth (Figure 5D–F). In line with these oncogenic characteristics of MED24 in human lung cancer cells, we found that NSCLC patients with MED24 high expression had a lower survival rate (Figure 5G and Figure S4).

## 4. Discussion

Using mouse models, we demonstrated that *Erbb2* deficiency prevented lung tumor development induced by deletion of *Pten* and *Smad4* by de-dysregulation of these cancer-related genes. Overlapping with *ERBB2*-correlated genes in human lung tumors, we further identified a subgroup of ERBB2-regulated genes that might be conserved in human and mouse lung tumors. Functional analysis of the top *ERBB2*-correlated genes (e.g., *MED24*) suggested that overexpression of MED24, a subunit of the Mediator complex, might be required for the ERBB2 pathway to dysregulate gene expression and the consequent lung cancer development.

The Mediator complex is a master regulator of transcription in all eukaryotes by transducing signals from the transcription activators to transcription machinery [28]. This complex consists of MED proteins (MED1–31) interacting with CDK8/19 and CycC [29]. Given that cancer development is frequently associated with significant alterations in gene expression and the Mediator complex has a global impact on gene expression, different subunits of the Mediator complex have been linked to cancer development [28]. For example, MED23 is selectively required for Ras-active lung cancer cells [30]; MED19 was found to promote tumorigenesis of lung cancer using a xenograft mouse model [31]; MED28 expression is positively associated with poor outcome in breast cancer patients [32]; and MED1 and MED24 were also reported to cooperatively contribute to the growth of breast cancer cells [33].

Here, we demonstrate that the MED complex is involved in ERBB2-dependent lung tumor development. MED24 may be a critical gene required for cell growth of lung cancer cells. We not only identified that *MED24* is a conserved downstream gene of ERBB2 in mouse and human lung tumors but also showed that its knockdown attenuates cell growth of lung cancer cells. This suggests that MED24 may play a crucial role in ERBB2-dependent lung tumor development. The treatments of EGF or Lapatinib (Figure 5A–C) suggested that ERBB2 phosphorylation (p-ERBB2), besides altered ERBB2 expression (Figure 4), was also capable of regulating MED24. It also suggested that it could be a strategy to treat lung cancer patients with high expression of MED24 using the inhibitor of p-ERBB2 (e.g., Laptinib). In addition, MED24 expression is significantly induced in *Pten*^−/−^*Smad4*^−/−^ lung tumors (Table S2), whose ERBB phosphorylation was reported to be significantly induced [12]. Meanwhile, we noticed that the impact of *MED24* knockdown on lung cancer cell growth is limited. Considering that the MED complex is usually required for a global transcription in eukaryotes [28] and that the *MED24* (also called *TRAP100*) whole-body knockout mouse is embryonic lethal [34], these cells with high efficiency of *MED24* knockdown may be lethal. In the future, generation of MED24 floxed mice will be an attractive way to study the in vivo function of MED24 in lung tumor development or other diseases.

In summary, by taking advantage of genetic mouse models, we demonstrated that ERBB2 was required for lung tumor development in vivo. Mechanistically, the genome-wide analysis showed that ERBB2 was required for dysregulated gene expression during lung tumor development. The systematic correlation analysis identified the conserved *ERBB2*-correlated genes between mouse and human lung tumors. Functional analysis of MED24, the top candidate gene of ERBB2, established an exciting mechanistic link between EGFR signaling and the Mediator complex. Clinically, MED24 expression is associated with a poor survival rate of NSCLC patients, suggesting MED24 as a potential target for lung cancer patients, especially those with ERBB2 alterations.

## Supplementary Materials

The following are available online at https://www.mdpi.com/2073-4409/8/6/615/s1, Figure S1: Immunohistochemistry analysis of lung tumors in *Pten*^d/d^*Smad4*^d/d^ mice at 12 to 13 months of age. Scale bar, 200 µm. Figure S2: The workflow of identification of ERBB2-correlated genes. Figure S3: Western blot analysis of protein expression of ERBB2 and MED24 in mice at 12 to 13 months of age (*n* = 5). Image J was used to quantify the relative level of protein bands; Each protein band was compared to the first band in the same row, respectively. Dot plot figures (lower panel) were generated based on the quantifications of Western blot (upper panel). Figure S4: Approximately 5% of lung cancer patients have MED24 mRNA upregulation. This is from the TCGA Provisional database (mRNA Expression z-Scores (RNA Seq V2 RSEM); Enter a z-score threshold ± 2.5), and it is the cutoff for the patient groups used in Figure 5F. Table S1: Summary of mouse lung pathology. Table S2: DEGs identified in the microarray analysis (*Pten*^f/f^*Smad4*^f/f^ vs. *Pten*^d/d^*Smad4*^d/d^). Table S3: DEGs identified in the microarray analysis (*Pten*^d/d^*Smad4*^d/d^*Erbb2*^d/d^ vs. *Pten*^d/d^*Smad4*^d/d^). Table S4: DEGs identified in the overlapping of microarray (*Pten*^d/d^*Smad4*^d/d^*Erbb2*^d/d^ vs. *Pten*^d/d^*Smad4*^d/d^) and of microarray (*Pten*^f/f^*Smad4*^f/f^ vs. *Pten*^d/d^*Smad4*^d/d^). Table S5: Upstream regulator analysis of DEGs identified in the overlapping of microarray (*Pten*^d/d^*Smad4*^d/d^*Erbb2*^d/d^ vs. *Pten*^d/d^*Smad4*^d/d^) and of microarray (*Pten*^f/f^*Smad4*^f/f^ vs. *Pten*^d/d^*Smad4*^d/d^). Table S6: ERBB2 positively correlated genes in TCGA lung adenocarcinoma. Table S7: ERBB2 positively correlated genes in TCGA lung squamous cell carcinoma. Table S8: ERBB2 negatively correlated genes in TCGA lung adenocarcinoma. Table S9: ERBB2 negatively correlated genes in TCGA lung squamous cell carcinoma. Table S10: ERBB2 positively and potentially regulated genes between human lung adenocarcinoma and mouse lung tumors. Table S11: ERBB2 positively and potentially regulated genes between human squamous cell carcinoma and mouse lung tumors. Table S12: ERBB2 negatively and potentially regulated genes between human lung adenocarcinoma and mouse lung tumors. Table S13: ERBB2 negatively and potentially regulated genes between human squamous cell carcinoma and mouse lung tumors.
