# Supplementary material for: ERBB2 Regulates MED24 during Cancer Progression in Mice with Pten and Smad4 Deletion in the Pulmonary Epithelium
Source: Cells. 2019 Jun 19;8(6):615. doi: 10.3390/cells8060615 (PMC6627404; doi:10.3390/cells8060615)
Supplement: Supplementary file 1 [file cells-08-00615-s001.zip › Cells-515655-supp-final/cells-515655-suppl-figures-FINAL-1.pdf]

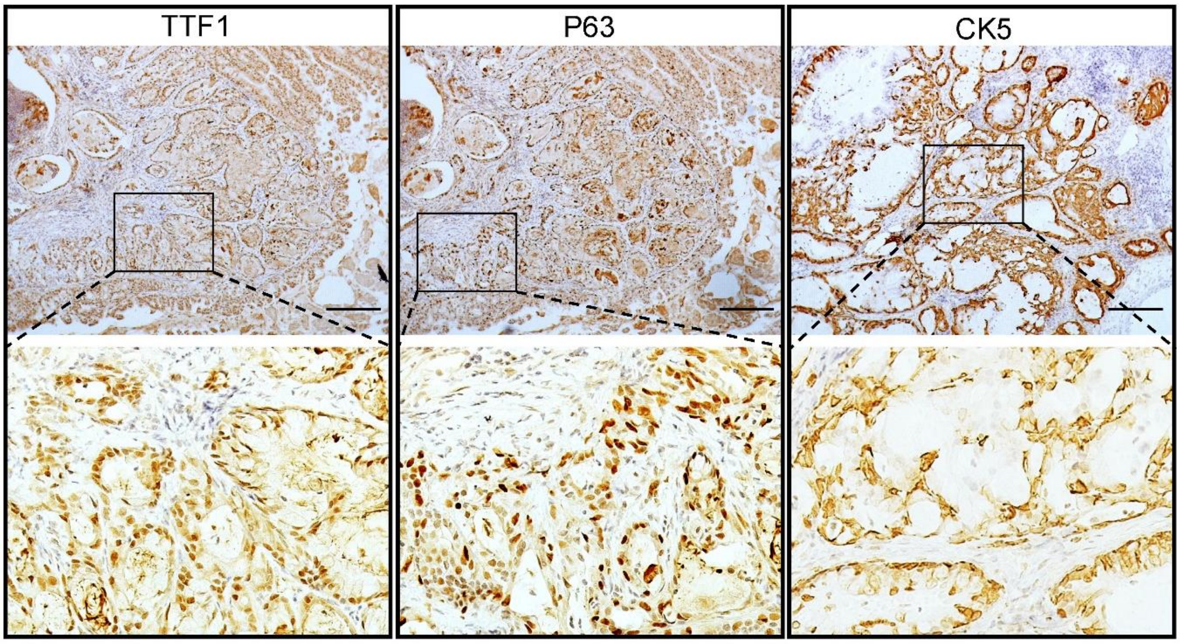

Figure S1. Immunohistochemistry analysis of lung tumors in *Pten*$^{d/d}$*Smad4*$^{d/d}$ mice at 12–13 months of age. Scale bar, 200μm.

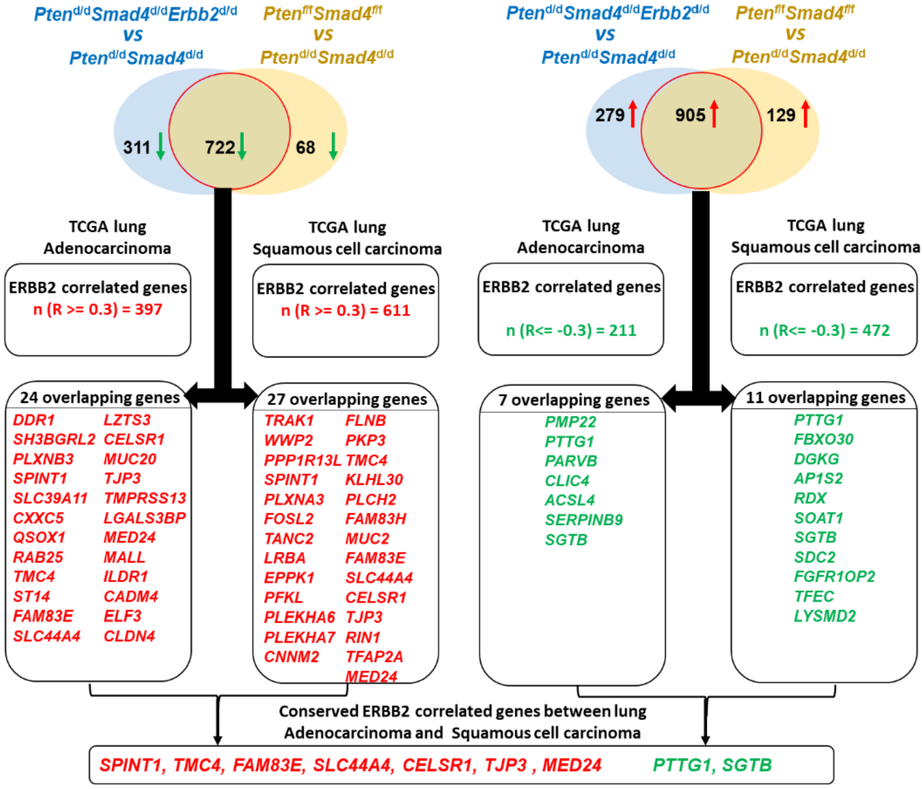

Figure S2. The workflow of identification of ERBB2 correlated genes.

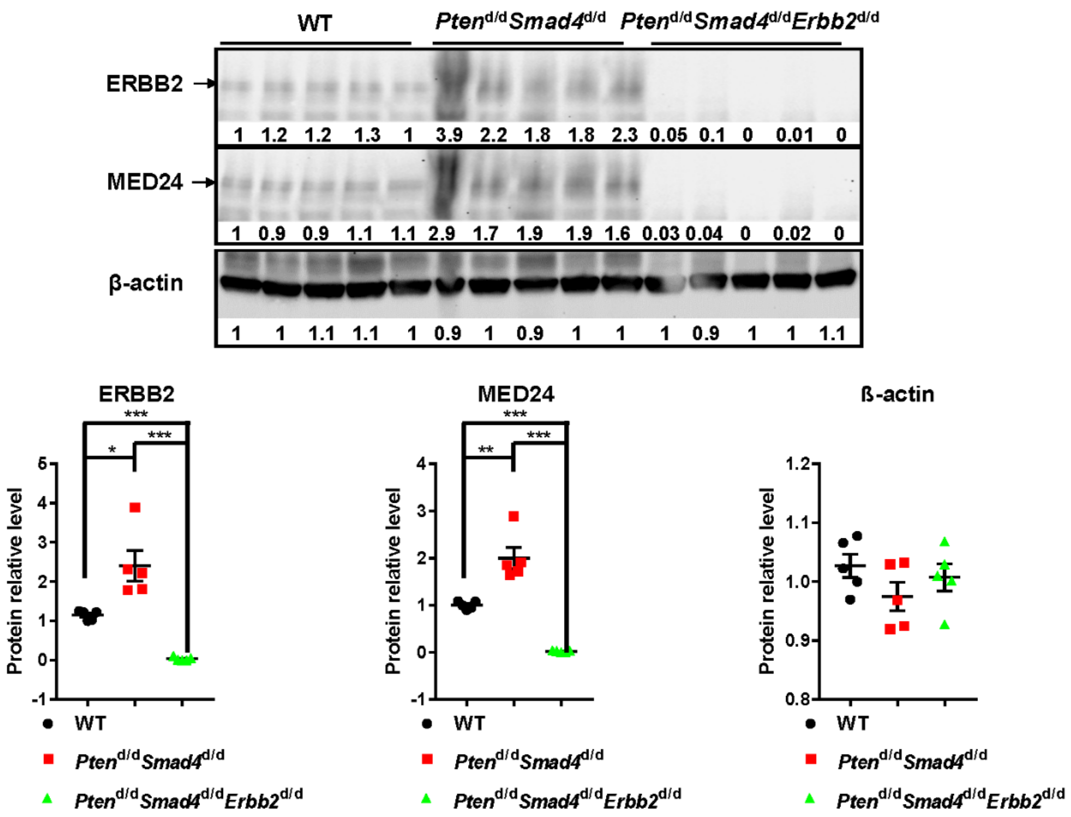

Figure 3. Western Blot analysis of protein expression of ERBB2 and MED24 in mice at 12–13 months of age (n = 5). Image J was used to quantify the relative level of protein bands; Each protein band were compared to the first band in the same row, respectively. Dot plot figures (lower panel) were generated based on the quantifications of Western Blot (upper panel).

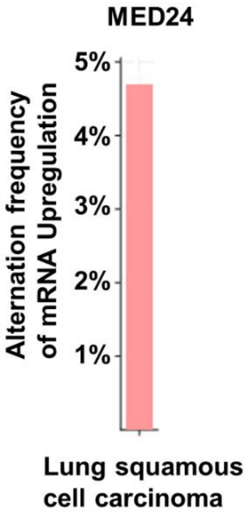

Figure S4. Approximately 5% of lung cancer patients have MED24 mRNA upregulation. This is from the TCGA Provisional database (mRNA Expression z-Scores (RNA Seq V2 RSEM); Enter a z-score threshold ± 2.5), and it is the cutoff for the patient groups used in Figure 5F.
